# When to initiate ECMO with low likelihood of success

**DOI:** 10.1186/s13054-018-2162-2

**Published:** 2018-09-19

**Authors:** Graeme MacLaren

**Affiliations:** 1 Cardiothoracic Intensive Care Unit, National University Health System, 5 Lower Kent Ridge Rd, 119074 Singapore, Singapore; 2 Department of Paediatrics, Paediatric Intensive Care Unit, University of Melbourne, The Royal Children’s Hospital, Flemington Rd, Parkville, VIC 3052 Australia

**Keywords:** Extracorporeal life support, Futility, Bone marrow transplant, Adult

Over the past decade, extracorporeal membrane oxygenation (ECMO) has become mainstream therapy in adult intensive care. The annual number of adult ECMO patients reported to the Extracorporeal Life Support Organization (ELSO) Registry overtook the number of neonatal and paediatric patients in 2012–2013 and now constitute the majority of cases [1]. Over time, the list of contraindications to ECMO has shrunk considerably and few of these remain absolute [2]. There are a number of circumstances outside of this list of contraindications to which ECMO could be applied but which constitute a very high-risk group with a low likelihood of success. This may be due to limited effective treatment for the underlying disease; the inherent fragility, size or age of the patient; or limitations in institutional resources and experience. Examples of such underlying diseases are disseminated herpes simplex virus or *Bortedella pertussis* pneumonia in young infants, both of which are associated with in-hospital survival rates of approximately 25–30% with ECMO [3, 4]. Some world-class institutions do not offer ECMO for children with these infections on the grounds of futility. Interestingly, these figures are comparable to the survival rates generally seen in adult ECPR (extracorporeal cardiopulmonary resuscitation) for in-hospital cardiac arrest [1, 5], which is usually not regarded as futile. Extremes of age are associated with similar hazards. Babies less than 34 weeks gestational age or < 2 kg are at higher risk of adverse outcomes, as are patients over the age of 70 years [6]. Even poorer outcomes may be expected in other instances. For example, fewer than 20% of those who receive ECMO for acute respiratory failure after hematopoietic stem cell transplantation will survive to hospital discharge [7, 8]. Indeed, refractory respiratory failure in the first 6 months after allogeneic bone marrow transplantation is still widely regarded as an absolute contraindication to ECMO, with survival < 5%. Nonetheless, some patients are now surviving these conditions, although there are vanishingly few of them. Less than 10–20% survival may sound dismal from the perspective of the treating institution and may be used to justify abandoning similar future rescue attempts, but the survivors themselves are unlikely to support this approach, if anyone were to ask them.

When should ECMO not be offered—where in the sand should the line be drawn? Conditions with expected survival rates of < 30%? < 10%? In attempting to answer this question, it should first be acknowledged that survival to hospital discharge is not the most important outcome measure, but rather ‘good long-term survival’—adequate neurological, psychological and functional recovery coupled to an acceptable quality of life, recognizing that there are many plausible definitions and subjective components of this. To date, insufficient attention has been given to systematically assessing long-term outcomes in ECMO survivors, although this has begun to change in some parts of the world. For example, Holland has a nationwide, government-funded programme where neonatal and paediatric ECMO survivors are comprehensively assessed at regular intervals by a multidisciplinary healthcare team for up to 18 years after ECMO [9] and some important research is emerging from this [10]. Nothing comparable yet exists for adult ECMO survivors. The potential impact of this on the decision to cannulate is self-evident: If 30% of patients with a given disease survive hospitalization after ECMO but most die within the next 12 months or are left with severe neurological damage and poor quality of life, this is quite different than if all the survivors return to their baseline level of functioning and go on to lead fulfilling lives.

Second, intensive care clinicians should not regard themselves as the sole arbiters of resource allocation and thus decide who receives or is denied ECMO based on a hazy, imprecise prediction of the patient’s chances of survival coupled to a potentially misguided sense of distributive justice [11]. In other words, we ought to be careful not to deny an equivocal ECMO candidate a chance at recovery only on the grounds that this might be unfair to other potential ECMO patients with a higher likelihood of survival. Such an acknowledgement should not be used to justify putting every patient indiscriminately on ECMO but neither does it support outright rejection of high-risk candidates in every instance. In addition to the clinician’s view as to the likely short-term outcome, other variables which should be considered include institutional experience, resources and policies; discussions with the patient’s next-of-kin; an assessment of the likely long-term survival, function and quality of life; and the availability and quality of rehabilitation, aftercare and home support, which vary enormously across different parts of the planet.

Lastly, if clinicians always reject high-risk ECMO candidates, they will never learn whether some of them could ever have been saved, given more time or new management strategies. For example, 25 years ago it was common practice to withdraw care in venovenous ECMO patients after 14 days if there was no evidence of lung recovery. This approach is flawed—the patient should continue to receive venovenous ECMO until they recover, require lung transplantation, or suffer a defining, life-limiting complication. To date, the longest ECMO run without recourse to transplantation and with good functional recovery is 605 days (Dr R. H. Bartlett, personal communication). In the words of T. S. Eliot, “Only those who will risk going too far can possibly find out how far it is possible to go.”

Nonetheless, the downside to offering ECMO to patients who are unlikely to survive is obvious. It may prolong the suffering of the patient and family and can exact a heavy toll from the ECMO team, in particular the nursing staff, who may regard ECMO under such circumstances as tragic, futile, misguided or harmful.

Until it can be established with greater certainty what the likelihood of ‘good long-term survival’ is by conducting comprehensive follow-up studies of ECMO patients, what can be done today when the next high-risk ECMO patient is referred? It is often easier to say “no” than “yes”, but we should not always opt for the path of least resistance (Table 1). Choosing to offer ECMO in such circumstances is predicated on serving the patient’s best interests and seeks to balance the benefits of attempting a heroic rescue versus the risks of providing futile care and prolonging suffering. These are difficult decisions, need to be tailored to individual patient and institutional circumstances and are often best not made alone. Thankfully, there is often time to decide in advance whether a given patient will be offered ECMO in the event they deteriorate. Discussions with ICU colleagues, both senior and junior, and liaising with ECMO directors in national or international centres experienced in managing specific conditions may be fruitful. Without abrogating individual responsibility, embarking on a plan of action as a group may help avoid resentment, miscommunication or abrupt changes in management during the subsequent ECMO run. If ECMO is offered to very high-risk patients, ensuring that the patient’s family as well as the entire ECMO team know why this strategy has been chosen and what the possible outcomes are may help everyone involved prepare for the worst, while they work toward and hope for the best.

**Table 1:** Checklist prior to initiating high-risk ECMO^a^

1. Is long-term survival with adequate neurological and functional recovery conceivable?	
2. Does the institution currently have sufficient resources and expertise? If not, is referral to another centre feasible?	
3. Is the institution ready to offer long-term support after ECMO, e.g. protracted ICU stay, transplantation, home ventilation?	
4. Is the patient’s family fully informed of the risks, do they understand the likely outcome and are they nonetheless supportive?	
5. Is the ECMO leadership within the institution supportive?	
If the answer to any of these questions is “no”, then ECMO should be reconsidered.	

*ICU* intensive care unit

*ECMO* extracorporeal membrane oxygenation

^a^ECMO which is not actively contraindicated but where survival to hospital discharge is unlikely
